# The design and testing of a novel mechanomyogram-driven switch controlled by small eyebrow movements

**DOI:** 10.1186/1743-0003-7-22

**Published:** 2010-05-21

**Authors:** Natasha Alves, Tom Chau

**Affiliations:** 1 Bloorview Research Institute, Bloorview Kids Rehab, Toronto, Ontario, Canada; 2 Institute of Biomaterials and Biomedical Engineering, University of Toronto, Toronto, Ontario, Canada

## Abstract

**Background:**

Individuals with severe physical disabilities and minimal motor behaviour may be unable to use conventional mechanical switches for access. These persons may benefit from access technologies that harness the volitional activity of muscles. In this study, we describe the design and demonstrate the performance of a binary switch controlled by mechanomyogram (MMG) signals recorded from the frontalis muscle during eyebrow movements.

**Methods:**

Muscle contractions, detected in real-time with a continuous wavelet transform algorithm, were used to control a binary switch for computer access. The automatic selection of scale-specific thresholds reduced the effect of artefact, such as eye blinks and head movement, on the performance of the switch. Switch performance was estimated by cued response-tests performed by eleven participants (one with severe physical disabilities).

**Results:**

The average sensitivity and specificity of the switch was 99.7 ± 0.4% and 99.9 ± 0.1%, respectively. The algorithm performance was robust against typical participant movement.

**Conclusions:**

The results suggest that the frontalis muscle is a suitable site for controlling the MMG-driven switch. The high accuracies combined with the minimal requisite effort and training show that MMG is a promising binary control signal. Further investigation of the potential benefits of MMG-control for the target population is warranted.

## Background

Individuals with severe physical disabilities often use access technologies as an alternative means of communication, environmental control or computer access. By providing a switching interface that the user is capable of controlling, access technologies promote an individual's independence and participation in daily living tasks [1]. Depending on the user's physical abilities, switching interfaces may range from simple mechanical buttons to brain-computer interfaces [2]. Often, individuals who are severely disabled may retain the ability to contract certain muscles. For example, individuals with high-level spinal cord lesions may have sufficient muscle control to move their head [3], and may therefore be able to use mechanical head-switches, tilt switches [4], or head-operated joy-sticks [5]. In cases where the individual lacks a high degree of motor function, an alternative solution is to use the remaining contractile ability of muscles.

Conventional muscle-based devices are controlled by electromyogram (EMG) signals from viable muscle sites of the hand, foot, cheek or forehead [6,7], and are commercially available (eg. The Impulse™ Switch by AbleNet^®^). The advantage of using muscle activity as the switching control for access devices is that physical movement is unnecessary, enabling the user to control the device even when only weak volitional muscle activity exists. Further, once the muscle site is located and the sensor is attached to the skin, switch performance is not compromised by misalignment of switch position due to body movements. This is an advantage over non-contact switches controlled by physical movement, such as infrared detectors (ex. IST switch by Words+^®^), optical detectors [8], or vision-based movement detectors [9,10], that are sensitive to the position of the sensor with respect to the access site on the body.

In addition to exhibiting changes in electrical activity detected by EMG, a contracting muscle also shows changes in its mechanical activity. The mechanical index of muscle contraction is known as the mechanomyogram (MMG). MMG is generated from gross lateral movement of the muscle at the initiation of a contraction, smaller subsequent lateral oscillations at the resonant frequency of the muscle, and dimensional changes of active muscle fibers [11-13]. MMG may be measured by microphones [14], piezoelectric contact sensors [15,16], accelerometers [17] or laser distance sensors [18] on the surface of the skin. Although MMG has found important applications in the assessment of muscle pathologies such as pain [19], fatigue [20,21] and disease [22], it has been under-studied as a control signal for alternative access. MMG may offer several advantages over conventional EMG muscle monitoring. It provides a better estimation of the inflection points in motor-unit recruitment and firing rate [23]. Since it is a mechanical signal, it is not influenced by skin impedance changes and does not require skin preparation. This makes it suitable for monitoring muscles when the overlying skin is prone to perspiration. Because MMG is typically measured by a single small sensor, it occupies a smaller footprint on the skin than differential EMG electrodes, making it suitable for non-invasive monitoring of smaller muscles. The single-sensor measurement is not dependent on the alignment along the muscle fibre axis, and is therefore less prone to faulty signal recordings when the user or caregiver may be unfamiliar with muscle anatomy. In addition, since MMG sensors are reusable, once purchased, they may be less expensive than disposable EMG electrodes. Because of these potential advantages, MMG has been investigated as a control signal for upper-limb prostheses [24,25] and powered orthotic devices [26]. Offline pattern recognition methods have shown that multi-site MMG signals are discernable during different patterns of forearm muscle contraction [27,28], indicating that MMG may find applications in multifunction control of access devices.

In this study we demonstrate an MMG-based binary switch and test its performance in detecting contractions of the frontalis muscle during small eyebrow movements. It has previously been reported that eyebrow movements may be used as a switch for users with pervasive motor impairments [8]. Although binary switches have limited functionality, they are of profound importance in enabling individuals with severe disabilities to achieve interaction with, and control of, their environment. By enabling the user to activate toys, speech output systems, light displays, and computer access via scanning keyboards, binary switches help the individual to overcome barriers to access.

The challenge in the design of an MMG-driven switch is to reliably convert the MMG signal into a switch-activation signal. To this end, we describe a real-time wavelet-based contraction detection algorithm in sections A-D. The switch is designed to harness small contractions of the frontalis muscle in real-time, while being resilient to artefact such as eye-blinks and head movements that commonly compromise the MMG signal. In sections E and F, we describe tests on able-bodied individuals to demonstrate the real-time performance of the detection algorithm, assessed in a single-switch paradigm, when user-dependent errors are minimal. We further examine the accessibility of the MMG switch by testing it on an individual with severe physical disabilities. The paper concludes with a presentation and discussion of the empirical results.

## Methods

### A. Instrumentation

MMG was measured by a microphone-based sensor manufactured according to the method of Silva et al. [29]. A program was written in LabView to perform real-time data acquisition, contraction detection and switch activation. Microphone-detected MMG signals were continuously sampled at 1 KHz (NI USB-6210, National Instruments). The LabView program allowed online modification of parameters such as switch debounce time and activation thresholds, and provided the user with visual and auditory feedback when a muscle contraction was detected. On detecting a contraction, the DTR pin on a serial port of the computer was asserted. The serial port was interfaced with a conventional 1/8" mono-plug via an opto-isolator (4N36, Motorola Inc) to provide a standard switch output. A keyboard interface (KE-USB36, Hagstrom Electronics) was used with the mono-plug for computer access.

### B. Contraction detection algorithm

Microphone signals were band-pass filtered with a 5^th ^order Butterworth filter with a cut-off frequency range of 5-100 Hz. The low cut-off attenuates the effects of movement [30], while the high cut-off attenuates any noise beyond the accepted MMG signal range.

The contraction detection algorithm used in this study is a modification of the off-line activity-detection algorithm proposed by Alves and Chau [31]. In this study, continuous-wavelet-transform (CWT) coefficients of the MMG signal are compared to scale-specific thresholds to identify voluntary muscle activity of the frontalis muscle during small eyebrow raises. The CWT is defined as(1)

where *x*_*mmg *_is the filtered MMG signal, and *ψ *is a mother wavelet shifted by *k *and scaled by *a *(*k*, *a *∈ ℜ).

In the contraction-detection scheme, CWT transform coefficients at 14 scales, *a*, were compared to scale-specific thresholds, *h*(*a*), derived from baseline recordings. A muscle contraction event, *z*, is detected at sample *k *when the coefficients of at least *j *scales exceed their thresholds, i.e.(2)

and(3)

where K_*baseline *_are the samples corresponding to the baseline MMG signals and *γ *is the threshold-scaling factor.

The scaling-factor *γ *could be varied between 1.2 and 2.5 in increments of 0.2. The value of *j *was set to 1. CWT analysis was performed on 100 ms long MMG signals, using the sym7 mother wavelet at scales with pseudo-frequencies that spanned the 5-100 Hz frequency range of interest, i.e. *a *∈ {7,9,10,12,14,15,17,20,23,28,35,46,69,115}.

### C. Post processing, noise detection and switch debouncing

**Figure 1:**
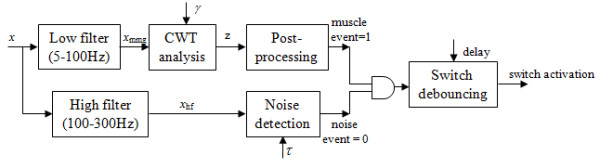
**Switch activation scheme**. Here x, x_mmg _and x_hf _are the microphone, MMG and high-frequency filtered signals, respectively; *γ *is the threshold scaling factor; *z *is the muscle-contraction event signal; and *τ *is the threshold that separates contraction from sensor movement.

Figure 1 shows the procedure for converting the continuously acquired microphone signal, *x*, to a switch activation signal. CWT analysis was performed on the MMG signal, *x*_*mmg*_, using non-overlapping sliding windows, 100 ms in length. The output of CWT analysis is a muscle activity event, *z *[*k*], for each sample, *k*, of the windowed MMG signal. To reduce the probability of spurious activity being detected as voluntary contractions, when fewer than 10 ms of activity was detected in the 100 ms window, the activity was not considered a valid muscle event, i.e.(4)

where *m *is the current window, and *K *= 100 is the window size.

**Figure 2:**
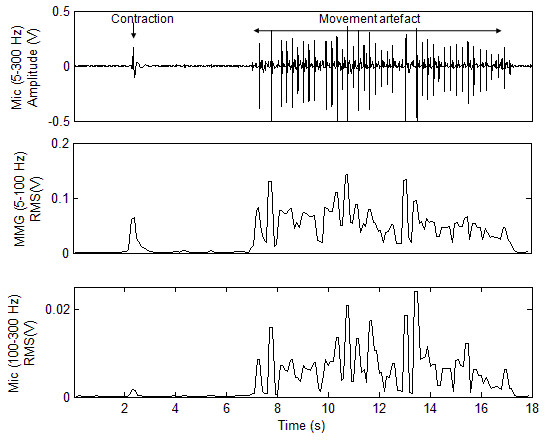
**Signal denoising**. The microphone signal and RMS values of the low-frequency (MMG) and high-frequency filtered signals during contraction and movement.

CWT coefficients of MMG signals during eyebrow movement exceed those of artefact such as eyeblink and head movement. However, high-amplitude artefacts are observed in the MMG signal when the sensor is being moved during activities such as donning, doffing or adjusting the sensor position. While both contractions and movement are detected in the microphone signal associated with MMG (5-100 Hz), movement is more prominent and differentiable in the high-frequency microphone signal (100-300 Hz). Figure 2 shows an example of the low-frequency (MMG) and high-frequency components of the microphone signal during muscle contraction and sensor movement. The RMS of the high-frequency signal, *x*_hf_, shows good separation during contraction and sensor movement, and was therefore used to detect noise, *n*, at each window *m *of length *K *= 100 samples, i.e.(5)

where threshold *τ *is determined from the maximum RMS of *x*_hf _during contraction. The noise event indicator was asserted if noise was detected in any of the *M *preceding windows, i.e.(6)

In this implementation *M *was set to 5, thus disabling the switch if noise was detected in the preceding 500 ms.

The switch was enabled when a muscle event was detected and a noise event was absent. To avoid single contractions that typically last longer than 100 ms from being converted to multiple switch activations, the switch output was debounced with an adjustable delay. The delay was dependent on the speed at which the user could comfortably raise their eyebrow, and could be adjusted between 100-600 ms in 100 ms increments.

### D. Events included in the baseline signal

**Figure 3:**
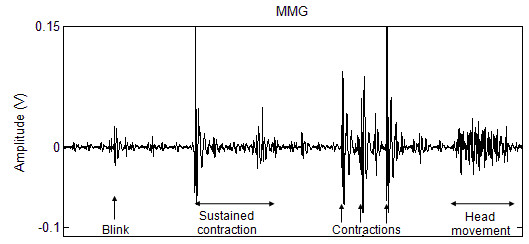
**Typical MMG signal recorded from the frontalis muscle during quick and sustained eye-brow raises, eye blinks and head movement**.

The performance of the detection algorithm is profoundly affected by the choice of thresholds, and hence, the baseline signal that encompasses the artefact expected during switch use. Even when the forehead is at rest, the MMG signal recorded at the frontalis muscle is affected by visually-observable periodic artefact due to blood flow. As seen in Figure 3, the signal is further compromised by artefact due to eye-blinks and head movement. The characteristic MMG signal when the eyebrow is raised is an oscillatory wave whose amplitude initially rises and then decays. While the high amplitude at the initial burst of activity facilitates the detection of contraction onset, the eventual decay in activity encumbers activity-detection during sustained contractions. This limits the potential of a secondary switch activated by sustained eyebrow raises.

**Figure 4:**
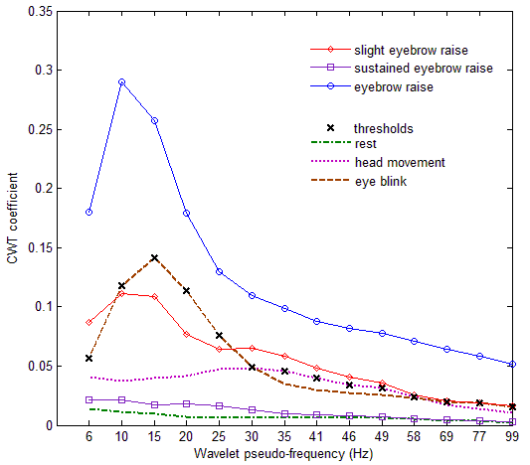
**Typical CWT coefficients of MMG recorded at the frontalis muscle**. The maximum coefficients at 14 scales are shown for different contraction conditions. The dashed lines depict CWT coefficients of the artefact in the MMG signal during rest, eye blinks and head movements. The maximum coefficients across the artefacts are the scale-specific thresholds (x) for contraction-detection. The solid lines depict coefficients for the events to be detected. Contractions are detected when the CWT coefficient of at least one scale is higher than the threshold. After the initial signal transient, sustained contractions could not be detected.

Figure 4 shows the maximum coefficients of the MMG signal during events such as rest, eye-blink, head movement, quick eyebrow raises and sustained frontalis contractions. The scale-specific thresholds of the detection algorithm are derived from the maximum coefficient of baseline MMG signals at each scale. The baseline includes MMG recorded during rest, blink and head movement. A contraction is detected if the CWT coefficient of at least one scale exceeds its baseline-derived threshold. The coefficients of the steady-state MMG during sustained contractions, while higher than the coefficients during rest, are confounded by those during movement artefact; therefore, sustained muscle activity cannot be detected. The signal transient at the initiation of contraction, however, has sufficiently high CWT coefficients to facilitate contraction-detection even during low-effort eyebrow raises. A quick and small contraction was therefore chosen as the preferred method for switch activation.

The detection algorithm was evaluated in real-time to monitor voluntary activity of the frontalis muscle and to generate a switch output.

### E. Protocol for performance testing

A convenience sample of ten able-bodied individuals (5 male), age 27 ± 2 years, provided written consent to participate in the study. These participants, referred to as A1-A10 in this study, had no previous history of musculoskeletal illness. An adult with C1-C2 incomplete spinal cord injury (SCI), referred to as B1, was also recruited. B1's method of access included a sip-and-puff switch for wheelchair control, a head tracker (TrackerPro^®^, Madentec) for computer mouse emulation, and the dwell function (250 ms) of the head tracker for emulation of a mouse click.

**Figure 5:**
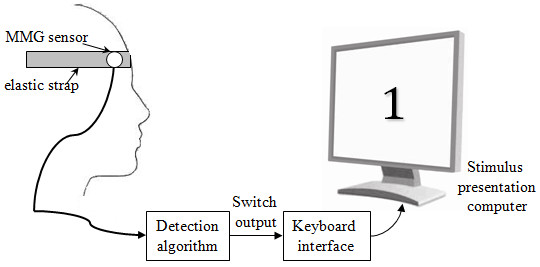
**Schematic diagram of equipment set-up**.

Participants were instrumented with an MMG sensor [29] attached to the frontal belly of the occipitofrontalis muscle of the forehead with an elastic strap, as shown in Figure 5. The sensor was placed 1 cm above the eyebrow, above the inside corner of the right eye. Once the sensor was affixed, participants performed 30 s of 'baseline' activities such as blinking, talking, smiling and moving their head. Scale-specific thresholds were automatically evaluated from the baseline MMG signals using the contraction-detection software written in LabView. The threshold scaling factor was selectable in the 1.2-2.5 range, and was adjusted for each participant such that false activations due to blinks and movement were avoided and participants were able to activate the switch by raising their eyebrows with minimal effort. Once participants demonstrated that they could perform 10 consecutive cued switch activations correctly, the threshold parameters were set and remained unchanged for the remainder of the experiment.

Custom switch assessment software was written in Visual Basic to present participants with audio-visual stimuli and to record the times of switch activation and stimulus presentation. Participants were presented with a pseudo-random sequence of numbers at 2 s intervals, and were asked to activate the switch by raising their eyebrows slightly when the number "1" was presented. Participants performed four trials of the experiment, with a 30 s break in between trials. One-hundred stimuli were presented during each trial, with the actionable stimulus (i.e. number 1) being presented 25% of the time. Throughout the session, participants were encouraged not to sit absolutely still, but rather to behave in a manner that they normally would when seated at a desk: they were free to blink, sway their chair slightly, move their head and talk without moving their eyebrows or the strap. The number of true positives (TP), true negatives (TN), false positives (FP) and false negatives (FN) were recorded during the cued stimulus tests.

In addition to responding to cued stimuli, participant B1 typed a pangram for each of two selection modalities: dwell and eyebrow-raise. For both typing tasks, B1 used the head-tracker to point to a character on an on-screen keyboard. For the first task, B1 dwelled at the character's location for 250 ms to select it; this was the method B1 regularly used for typing for more than seven years. For the second task, B1 raised his eyebrow to select the character. The time taken to complete each task was recorded.

After the data-collection trials were completed, all participants practiced using the switch for 1 hour, performing activities such as typing using a scanning keyboard. At the end of the hour, participants were asked to rate the level of effort and fatigue associated with controlling the eyebrow switch on a five-point linear scale: [1-Nothing at all, not tired; 2- A little, not tired; 3- Moderate, a little tired; 4- A lot, tired; 5-Too much, very tired]. In addition, participants were asked to rate if they had to try multiple times before activating the switch: [1-Never; 2- Very infrequently; 3- Sometimes; 4- Very often; 5- Almost all the time].

The experimental protocol was approved by the hospital and university research ethics boards, and was in compliance with the Declaration of Helsinki.

### F. Performance Metrics

The sensitivity and specificity of the MMG switch were evaluated from the cued stimulus test, and are given by(7)

and(8)

Sensitivity is a measure of correctly identified muscle contractions, while specificity is a measure of correctly rejected artefacts.

Trends in response delay were used to gauge if participants were fatigued from prolonged use of the eyebrow switch. For each participant, a linear regression of response delay against elapsed session time was evaluated, and the 95% confidence-interval (CI) of the slope was computed. Here it is assumed that response time increases with increasing fatigue.

## Results

The participant-chosen threshold scaling factor, *γ*, ranged from 1.5 to 2.3, and was dependent on what the participant perceived to be baseline noise. The switch performance metrics are shown in Table 1. The switch showed almost perfect sensitivity and specificity for all participants. As reported by the participants, activities such as batting eyelids or involuntary changing facial expressions sometimes resulted in false detections. Participants reported that multiple attempts to activate the switch were infrequent. When required, the multiple attempts usually included a very small contraction followed by a stronger contraction. On average, participants rated that switch activation required very little effort and was not tiring to use. The response time of only one participant (A10) had a small but significant (95% CI > 0) increase over the course of the experiment.

**Table 1:** Performance metrics for the eyebrow switch.

Participant	Contraction detection		Attempt rating	Effort rating	Slope of response time	
	Sensitivity	Specificity			95% CI of slope (ms/min)	
B1	1.000	1.000	1	2	-10.25	-1.75
A1	1.000	1.000	1	2	-4.17	-0.31
A2	1.000	1.000	1	2	-9.71	-0.11
A3	1.000	1.000	1	2	-0.15	6.77
A4	1.000	0.997	2	2	-4.19	4.92
A5	0.990	1.000	2	2	-1.59	2.55
A6	1.000	1.000	2	3	-5.83	3.57
A7	1.000	1.000	2	1	-4.36	5.35
A8	0.990	1.000	1	2	-14.64	-5.84
A9	0.990	0.997	2	3	-2.33	9.54
A10	1.000	1.000	1	2	5.84	11.69
						
Average	0.997 ± 0.004	0.999 ± 0.001	1.45 ± 0.5	2.1 ± 0.5	-4.67 ± 5.5	3.30 ± 5.1

Multiple attempt rating: Did you have to try more than once before activating the switch? [1-Never; 2- Very infrequently; 3- Sometimes; 4- Very often; 5- Almost all the time]

Effort rating: How much effort was required to activate the switch? [1-Nothing at all, not tired; 2- A little, not tired; 3- Moderate, a little tired; 4- A lot, tired; 5-Too much, very tired]

CI -confidence interval; Slope units: response time (ms)/elapsed experiment time (min)

For participant B1, the time required to complete the typing task with the dwell switch was 63 s, while that for the eyebrow switch was 54 s. No typing mistakes were made for either switch modality. In addition, B1 reported that he perceived the eyebrow switch to have a faster response-time than the dwell switch.

## Discussion

The CWT detection scheme showed very high sensitivity and specificity in a switch paradigm where activation was controlled by contractions of the frontalis muscle during eyebrow raises. CWT detection has been shown to have comparable sensitivity to RMS and absolute-value muscle-activity detectors, while outperforming these detectors in terms of specificity [31]. The MMG signal is non-stationary during sustained contractions [27], warranting the use of time-frequency analysis. The switch required minimal training, and only the threshold scaling factor needed adjustment before use. By using scale-specific thresholds that are dependent on the baseline signal, the detection scheme can estimate the noise level according to measurement conditions, and does not require the user to finely tune each threshold.

The primary function of the frontalis is to raise the eyebrow; hence, contraction of the frontalis often accompanies movement of the skin proximal to the eyebrow. Muscle-contraction detection has some notable advantages over conventional movement-controlled switches. First, commercially available non-contact movement-triggered switches (ex. IST switch by Words+^®^) are sensitive to the position of the transducer relative to the access site, and may pose safety hazards when the transducer is mounted by supports that are in close proximity to the eye. Second, movement-based detectors often require prominent movement, and hence, require more effortful muscle contractions which may be fatiguing for the user. This has been seen in the abandonment of an accelerometry-based access solution, where movement of a head-band during eyebrow raises was used for switch control [32]. The muscle-based switch, in contrast, required little effort for activation, as demonstrated by qualitative participant feedback and the trends in response time. Further, as a control site, the frontalis muscle is broad and has a large surface area on the forehead, thus offering flexibility with sensor placement.

The MMG signal is generated by the unfused mechanical activities of motor units. The bulk movement of the muscle and asynchronous activation of fibers at the initiation and end of contraction creates a high-amplitude transient that is easily detected. During a sustained contraction however, because of the fusion of motor unit activity [33], the differentiation between muscle activation and the resting signal may not be as obvious. Thus, fast muscle contractions may be more suitable for switch control than sustained muscle contractions where a prolonged 'ON' time may be difficult to detect, especially when the signal may be confounded by movement artefact. Since the ON time is sometimes used to control a secondary switch, this presents a limitation when compared to EMG-based switches (ex. The Impulse™ Switch by AbleNet^®^).

Microphones are less sensitive to motion artefact than accelerometers [34], and may be the preferred method for detecting MMG when the muscle site is prone to movement. Nonetheless, signal artefact during eye blinks and head movement, combined with the low-amplitude signal during sustained contractions, constrained us to use the signal transient for switch control. During eyebrow raises, the transient is often accompanied by skin movement, making it difficult to remove movement artefact using source-separation methods suggested for the decoupled microphone-accelerometer sensor employed in this study [29,35]. While we were able to overcome the false detection of contractions during head-sway and sensor movement by increasing the thresholds and analysing the high-frequency signal, artefact due to vigorous head movement, commonly seen in individuals with uncontrolled spasms or athetoid cerebral palsy, could not be removed or automatically identified. These confounding movements, however, affect a small portion of the population that could stand to benefit from this access technology. Movement artefacts could further be identified by analysing temporal patterns typical of the user's uncontrolled movement; however, this may result in longer switch response times, or may require additional instrumentation, such as tri-axis accelerometers.

As with other muscle-based control technologies [36], accuracy could likely be gained by using additional information available from larger windows of data. However, the speed-accuracy trade-off should be considered in the design of switching solutions. The delay introduced by the control system, which includes the time for acquiring data, processing data and actuating the device, should not be perceivable by the user: for upper-limb prostheses the acceptable delay is generally considered to be in the 200-300 ms range [36,37]. In its current implementation, the detection algorithm acquired and processed 100 ms of MMG data before generating a switch response. For the disabled participant, B1, although the time taken to complete the typing task with the eyebrow was only slightly less than that for the 250 ms dwell switch, the participant qualitatively perceived a significant reduction in response time. The appeal of active participation may have influenced this perception.

The performance metrics indicate that the individual with SCI could control the switch with accuracies comparable to that of able-bodied individuals. While the high sensitivity and specificity show the potential of the MMG as a reliable switch control signal, it is important to note that, for participant B1, the muscle site and its control were largely unaffected by the SCI. A limitation of this study is that it has not been trialed on individuals with neuromuscular disability at the access site. Non-verbal individuals with severe physical disabilities, due to conditions such as quadriplegic cerebral palsy, are often left without reliable access solutions and may therefore stand to benefit most from emergent access technologies. Control challenges posed when detecting activity in atypical muscles, and in discriminating between voluntary and involuntary activity when muscle control is compromised need to be further addressed and are deferred for future studies.

## Conclusion

An MMG-driven binary switch controlled by voluntary activity of the frontalis muscle has been proposed. The MMG-switch is designed to harness low-effort muscle contractions in real-time, while being resilient to artefact such as eye-blinks, head movements and sensor movements. The switch showed high sensitivity and specificity for cued response tests, was not fatiguing to use for prolonged periods, and required minimal effort to control. These results suggest that MMG may be used as a non-invasive access pathway for individuals who retain voluntary control of the frontalis muscle.

## Competing interests

The authors declare that they have no competing interests.

## Authors' contributions

NA designed and implemented the detection algorithm, designed the performance tests, performed data collection, analyzed the data, and drafted the manuscript. TC conceived the study, advised on the design and coordination of the experiments, and edited the manuscript. All authors read and approved the final version of the manuscript.
